# Revealing the impact of organic spacers and cavity cations on quasi-2D perovskites via computational simulations

**DOI:** 10.1038/s41598-023-31220-8

**Published:** 2023-03-17

**Authors:** Diego Guedes-Sobrinho, Danilo Neves Silveira, Luis O. de Araujo, Jônatas Favotto Dalmedico, W. Wenzel, Y. Pramudya, Maurício J. Piotrowski, Celso R. C. Rêgo

**Affiliations:** 1 Chemistry Department, Federal University of Paraná, Curitiba, 81531-980 Brazil; 2 Department of Physics, Federal University of Pelotas, PO Box 354, Pelotas, RS 96010-900 Brazil; 3 Institute of Nanotechnology, Karlsruhe Institute of Technology (KIT), Hermann-von-Helmholtz-Platz, 76344 Eggenstein-Leopoldshafen, Germany

**Keywords:** Chemistry, Energy science and technology, Materials science, Nanoscience and technology, Physics

## Abstract

Two-dimensional hybrid lead iodide perovskites based on methylammonium (MA) cation and butylammonium (BA) organic spacer—such as ${\hbox {BA}_{2}\hbox {MA}_{n-1}\hbox {Pb}_{n}\hbox {I}_{3n+1}}$—are one of the most explored 2D hybrid perovskites in recent years. Correlating the atomistic profile of these systems with their optoelectronic properties is a challenge for theoretical approaches. Here, we employed first-principles calculations via density functional theory to show how the cation partially canceled dipole moments through the ${{\hbox {NH}_{3}}^{+}}$ terminal impact the structural/electronic properties of the ${\hbox {Pb}_{n}\hbox {I}_{3n+1}}$ sublattices. Even though it is known that at high temperatures, the organic cation assumes a spherical-like configuration due to the rotation of the cations inside the cage, our results discuss the correct relative orientation according to the dipole moments for ab initio simulations at 0 K, correlating well structural and electronic properties with experiments. Based on the combination of relativistic quasiparticle correction and spin-orbit coupling, we found that the MA horizontal-like configuration concerning the inorganic sublattice surface leads to the best relationship between calculated and experimental gap energy throughout n = 1, 2, 3, 4, and 5 number of layers. Conversely, the dipole moments cancellation (as in BA-MA aligned-like configuration) promotes the closing of the gap energies through an electron depletion mechanism. We found that the anisotropy $\rightarrow$ isotropy optical absorption conversion (as a bulk convergence) is achieved only for the MA horizontal-like configuration, which suggests that this configuration contribution is the majority in a scenario under temperature effects.

## Introduction

Even though the power conversion efficiencies (PCE) of three-dimensional (3D) metal halide perovskites (MHPs) have exceeded 25% for FAPbI$_{3}$^1^ (FA = formamidinium) and 22% for MAPbI$_{3}$^2^ (MA = methylammonium), the long-term stability of these materials in solar cell devices is limited, especially because of the poor heat and moisture stability^3,4^. Two-dimensional (2D) MHPs have emerged as an alternative to their 3D counterparts not only due to their improved stability^5–7^, but also because of their versatility regarding good structural flexibility and tunability of optical properties^8–10^. Among the 2D MHP possibilities, some attention has been devoted to the ${\hbox {BA}_{2}\hbox {MA}_{n-1}\hbox {Pb}_{n}\hbox {I}_{3n+1}}$ system based on butylammonium (BA) as a large monovalent cation spacer of the ${\hbox {Pb}_{n}\hbox {I}_{3n+1}}$ inorganic sublattices^11–15^. However, this system has not yet been studied extensively, so atomistic approaches through computational simulations are powerful in clarifying essential properties for the design of optoelectronic devices, notably solar cells, light-emitting diodes, and photodetectors.

${\hbox {BA}_{2}\hbox {MA}_{n-1}\hbox {Pb}_{n}\hbox {I}_{3n+1}}$ perovskites belong to the Ruddlesden–Popper (RP) perovskite family^16–18^, in which the BA spacer is a linear four-carbon chain splitting the inorganic part based on n layers (defining the inorganic quantum-well thickness as corner-sharing ${\hbox {Pb}_{n}\hbox {I}_{3n+1}}$ octahedrons), in which for n $\ge 2$ the cuboctahedral cavity sites are occupied by MA cations. Solar cell devices based on this 2D-RP MHP have exhibited improved light and moisture stability compared to 3D MAPbI$_{3}$^6,19^, but their highest PCE did not reach more than 12.5%^20^. Stoumpos et al. have synthesized and isolated ${\hbox {BA}_{2}\hbox {MA}_{n-1}\hbox {Pb}_{n}\hbox {I}_{3n+1}}$ as 2D-RP for n = 1, 2, 3, and 4 layers, performing the respective characterization by single-crystal X-ray diffraction^12^. Additionally in subsequent studies, systems with n = 5, 6, and 7 were also isolated^13,21^, which showed the convergent behavior of band gap energies throughout 2.43 (n = 1), 2.17 (n = 2), 2.03 (n = 3), 1.91 (n = 4), 1.83 (n = 5), 1.78 (n = 6), and 1.74 eV (n = 7), i.e., with a clear tendency to reach 1.55–1.67 eV for n = $\infty$ as cubic, tetragonal, and orthorhombic bulks^22–25^. Although advances have been made, while it is well known that the 3D bulk optoelectronic properties (such as absorption coefficient and gap energy) are strongly influenced by local distortions and polymorphic contributions^26–30^, in-depth detailing of these correlations for 2D-RP MHPs is scarce.

Theoretical approaches based on density functional theory (DFT) joined with experimental works have suggested that local distortions and asymmetry within inorganic layers must control spin-splitting in 2D MHPs^31^, which associated with spin-orbit coupling (SOC) is responsible for the Rashba/Dresselhaus spin-splitting^32–35^, which is also observed in even lead-free 3D MHPs^29,30^. On the other hand, it is important to mention that, even in 3D MHPs, the correlation between long carrier lifetimes and the presence of the Rashba effect is still being debated^36–41^. Recently, charge separation and long carrier lifetimes have been associated with edge states of the ${\hbox {Pb}_{n}\hbox {I}_{3n+1}}$ layer stabilized by internal electric fields created by the MA alignment into cavity sites^42^. However, only the parallel MA alignment (with respect to the organic-inorganic interface) in ${\hbox {BA}_{2}\hbox {MA}_{n-1}\hbox {Pb}_{n}\hbox {I}_{3n+1}}$ 2D-RP MHP for n = 1, 2, and 3 is considered, so the BA role in the interface and its small dipole moment interacting with MA also needs to be explored. Additionally, it is worth mentioning that under temperature 2D-RP perovskites are complex, e.g., the triclinic $\rightarrow$ orthorhombic phase transition has been observed in a short temperature range of 250 K $\rightarrow$ 300 K^12,13,15^. Advancing deeper, recent studies have reported the existence of a dynamic/thermal polymorphism (T > 0 K) and non-dynamic/non-thermal polymorphism (T = 0 K). The first one is associated with thermal fluctuations of the ions about their equilibrium positions^43^, while the second one could be considered as low symmetry distortions at 0 K leading to an increase of the stability relative to the high symmetry configurations. Thus, understanding polymorphism without temperature effects in detail is an initial step towards correctly describing the structure of the system at higher temperatures, given that it has been reported for several organic and inorganic 3D perovskites^29,30^. At the same time, on average, in 3D MHPs MA cation is dynamic under high temperatures due to the rotation of the cations inside the cage^22,44,45^, which is also observed within molecular dynamics approach for 2D perovskites^46^. Therefore, the atomistic behavior of organic spacers and cations and their impact on the inorganic quantum well need to be investigated in such a way that computational simulations, even at 0 K are a vital step to advancing the knowledge about 2D-RP MHPs.

Herein, we investigate the structural and electronic properties of ${\hbox {BA}_{2}\hbox {MA}_{n-1}\hbox {Pb}_{n}\hbox {I}_{3n+1}}$ 2D-RP perovskites through the first principles calculations based on DFT by taking n = 1, 2, 3, 4, and 5 as the number of layers. A detailed structural characterization protocol was employed to describe the role of BA organic spacers and MA organic cations in the local structural motifs. We have combined spin-orbit coupling effects and relativistic quasiparticle correction (called DFT-1/2)^47^ to calculate the band gap energies and the optical absorption coefficients, which have provided results in good agreement with experimental works and with a computational cost comparable to standard DFT calculations^30,48^. In excellent agreement with experimental reports, our 2D-RP results clarify the BA-MA arrangement role, confirming the importance of exploring structural configurations in atomistic simulations as a predictive tool in the context of materials design.

## Theoretical approach and computational details

### Structural configurations

**Figure 1:**
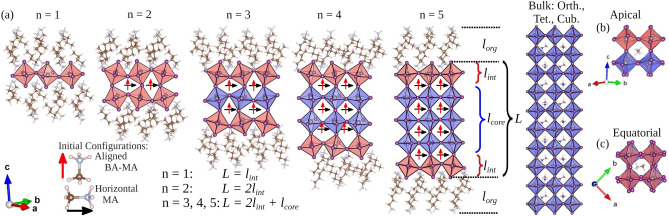
(**a**) Representation of ${\hbox {BA}_{2}\hbox {MA}_{n-1}\hbox {Pb}_{n}\hbox {I}_{3n+1}}$ 2D-RP perovskites with n = 1, 2, 3, 4, 5, and $\infty$ (orthorhombic, tetragonal, and cubic bulks). $l_\text {org}$ depicts the thickness of the BA cation-spaced region, where *L* is the inorganic layer thickness defined by $l_\text {int}$ and $l_\text {core}$ as interface and core octahedrons, respectively. The representations define the initial configurations based on MA horizontal-like (black arrow “$\rightarrow$”) and BA-MA aligned-like (red arrow “$\uparrow$”), whereas the cubic and tetragonal configurations were considered for the bulks (n = $\infty$). All 2D and 3D perovskites were structurally analyzed considering apical (**b**) and equatorial (**c**) orientations separately.

For $\text {n} \ge 2$ in ${\hbox {BA}_{2}\hbox {MA}_{n-1}\hbox {Pb}_{n}\hbox {I}_{3n+1}}$ 2D-RP perovskites, the presence of MA cations implies permanent dipole moments in the inorganic layer region, whose spatial orientations into the cuboctahedral cavities are important. Based on this, we explored different configurations considering the dipole orientations between BA and MA, since BA presents a small dipole moment at the end of the organic chain with the dipole pointed toward the inorganic layers, which allows us to investigate their influence on electronic properties given the possibility of alignment with the MA dipole. ${\hbox {BA}_{2}\hbox {MA}_{n-1}\hbox {Pb}_{n}\hbox {I}_{3n+1}}$ systems for n = 1, 2, 3, 4, 5, and $\infty$ (cubic, tetragonal, and orthorhombic bulks) are depicted in Fig. 1. The configurations were named according to the MA dipole orientations after fully relaxing, such as: (i) on the equatorial, i.e., MA lying on the *ab* plane named by MA horizontal-like and depicted by the black arrow “$\rightarrow$”); (ii) apically oriented to *c* direction, given that after fully relaxing BA-MA configuration stands as aligned-like, which is represented by the arrow “$\uparrow$”. It is essential to mention that BA-MA aligned-like presents a partial alignment between MA (pointed to apical *c* direction) and the terminal ${{\hbox {NH}_{3}}^{+}}$ group from BA, consequently reaching a partial canceling of the BA-MA dipole moments. In this scenario, both MA horizontal-like, with relative dipole orientations as $\overrightarrow{\mu }\text {(MA)}\cdot \downarrow {\mu }\text {(BA)}$, and BA-MA aligned-like, so that $\uparrow {\mu }\text {(MA)}\cdot \downarrow {\mu }\text {(BA)}$, were a set of configurations obtained for the quasi-2D-RP perovskites after reaching the convergence criterion through the protocol described below.

**Figure 2:**
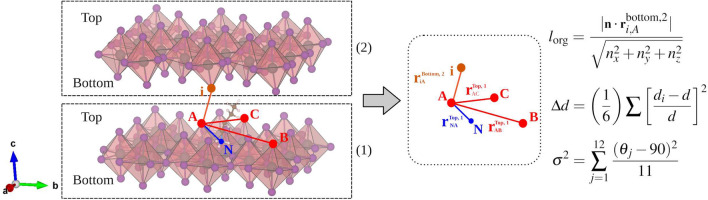
Inorganic layer space ($l_\text {org}$) calculated through ${{\textbf {n}}} = (n_x,n_y,n_z)$ as the normal vector with respect to the plane formed by the apical iodine atoms in the interface region on the top of the inorganic layer, i.e., ${{\textbf {n}}} = {{\textbf {r}}}_{AB}^{\text {top,1}}\times {{\textbf {r}}}_{AC}^{\text {top,1}}$ (index 1 is the inorganic layer of the plane equation). ${{\textbf {r}}}_{i,A}^{\text {bottom,2}}$ is the iodine vector position on the bottom of the opposite interface region (index 2). For bond length distortion ($\Delta d$), $d_i$ is the individual PbI bond distance and *d* is the mean distance of the six PbI bond forming the octahedra. The bond angle variance ($\sigma ^2$) is calculated through the diference the right angle (${90}{^{\circ }}$) and the individual I–Pb–I angles ($\theta _i$).

The thickness of the organic ($l_\text {org}$) and inorganic (*L*) regions was quantified in order to investigate the effects from the BA-MA configurations. Figure 1a shows *L* for the inorganic layer thickness composed of the interface octahedrons ($l_\text {int}$) in contact with the organic spacers and the core octahedrons ($l_\text {core}$) without contact with the organic layer, as the interface octahedrons surround them. The organic region space ($l_\text {org}$) was quantified, as well as the distortion degree from the octahedrons through the bond length distortion ($\Delta d$)^49–51^ and the bond angle variation ($\sigma ^2$)^49,52^ parameters, such as detailed in Fig. 2. Thus, the inorganic layer thickness is calculated as $L = l_\text {int}$ and $L = 2l_\text {int}$ for n = 1 and 2, respectively, while for n = 3, 4, and 5 it is given by $L = 2l_\text {int} + l_\text {core}$. The $d_i$ are the six individual Pb–I bonds and *d* is the average Pb–I distance for each octahedron. For $\sigma ^2$, $\theta _j$ are the I–Pb–I angles for the eight quadrants into a particular octahedron. Both parameters were calculated for the core and interface regions of the inorganic layer so that non-zero values represent a distortion degree from a Platonic octahedron.

### Total energy calculations and geometry optimization

We employed DFT^53,54^ calculations through the semilocal formulation proposed by Perdew–Burke–Ernzerhof (PBE)^55^ for the exchange–correlation energy functional as implemented in the Vienna *Ab initio* Simulation Package (VASP)^56,57^. The projector augmented-wave (PAW)^58^ method, in the VASP implementation^56,57^, was employed to solve the Kohn–Sham (KS) equations, wherein fully relativistic calculations describe the core states. At the same time, the scalar-relativistic approximation by including spin-orbit coupling (SOC) treats the valence states. Thereby, the following electron configurations were considered for the valence electrons: H ($1s^1$), C ($2s^2$, $2p^2$), N ($2s^2$, $2p^3$), Pb ($5s^2$, $5d^{10}$, $6s^2$, $6p^2$), and I ($5s^2$, $5p^5$). The Brillouin zone integration was performed considering a ${{\textbf {k}}}$-point mesh of $6 \times 6 \times {\mathbf{Z}}$, where **Z** = 3 for n = 1 and 2, and **Z** = 2 for n = 3, 4, and 5. For cubic, tetragonal, and orthorhombic unit cells, we used a ${{\textbf {k}}}$-point mesh of $8 \times 8 \times 8$, $6 \times 6 \times 4$, $6 \times 4 \times 5$, respectively. The plane waves were expanded up to the cut-off energy of 500 eV for all 2D and 3D perovskites, obeying the total energy convergence criterion of ${1.0\times 10^{-5}}$ eV. Given the vital role of weak van der Waals (vdW) interactions, especially for organic chain spacers, the lattice constants and stress tensors were fully optimized considering the D3 correction, as proposed by Grimme et al.^59,60^ wherein Hellmann-Feynman forces were relaxed to values less than 0.010 eV Å$^{-1}$ on every atom.

### Optoelectronic properties

For band gap energies and optical absorption calculations through a computationally efficient approach, we employed the DFT-1/2 quasiparticle correction method^47^. For this step of our calculations, we have employed the Workflow Active Node (WaNo) DFT-VASP^61–63^ developed within the SimStack workflow framework. Through the workflow, we save time by automating and reducing protocol complexity, permitting the monitoring of multiple sets of calculations for independent DFT protocols (such as PBE+D3, PBE+D3-1/2, PBE+D3+SOC, and PBE+D3+SOC-1/2) for a different number of layers in 2D-RP perovskites. Notwithstanding that, it is essential to mention that the PBE+SOC-1/2 protocol has been successfully applied to pure and mixed 3D halide perovskites^29,30,48^, from which the band gap energies for several 3D perovskites compositions are obtained as very close to the experimentally reported values, under a computational cost into standard DFT level. Here, the PBE+SOC-1/2 protocol has been employed for 2D-RP MHPs. This method is based on the Slater transition technique (STT)^47,64,65^, in which the half-occupied atomic eigenvalue is equal to the negative value of the ionization potential.

Half-occupancy is implemented by a modified KS potential as $V_\text {mod,KS} = V_\text {KS}(\textbf{r}) - V_S(\textbf{r})$, where $V_\text {KS}(\textbf{r})$ is the standard KS potential and $V_S(\textbf{r})$ is the self-energy potential. The $V_S(\textbf{r})$ is approximately given by the difference between the KS potential for the neutral (all-electron, ae) and half-ionized (ae-1/2) atoms, trimmed by a step function $\Theta (\textbf{r}, CUT)$, in order to avoid the penetration of the self-energy Coulomb tails into neighboring atom sites,

1 $$\begin{aligned} V_S (\textbf{r})=\Theta (\textbf{r},CUT) \left[ V_{ae}(\textbf{r})- V_{ae-1/2}(\textbf{r}) \right] ~. \end{aligned}$$

The *CUT* parameter represents the self-energy potential range around elements with a significant contribution to the band edge states. Its value is obtained variationally by maximizing the energy gap as described in more details elsewhere^29,48^. Thus, since its contribution is the majority, half-occupancy was employed for the valence band by considering the 5*p* state from the I atom. Figure S1 in Supporting Information shows the good structural transferability of the variationally optimized *CUT* parameter (at $3.88a_0$) for perovskites, maximizing the energy gap without considering empirical parameters.

## Results

### Structural analysis

The lattice parameters calculated for all 2D-RP perovskites are in excellent agreement with the experimental reports. Tables S1, S2, and S3 in Supporting Information show a comparison with respect to the triclinic ($P{\bar{1}}$^15^) at 250 K $\rightarrow$ orthorhombic (*Cc*2*m*^12,13^, *Cmca*^15^) at 300 K phase transition for 2D-RP systems lying between n = 2 and n = 4, whereas for n = 5 it is an orthorhombic (*C*2*cb*^13^). Although there is a slight expansion in the triclinic to orthorhombic transition (less than 3 %) reported by experiments, our simulations involving the MA horizontal-like and BA-MA aligned-like configurations at 0 K present smaller percent deviations than 5 % in modulus for the lattice parameters (Table S1) and supercell volume (Table S2) in comparison with both triclinic and orthorhombic phases. Except for the orthorhombic (in which $\alpha = \beta = \gamma = {90}{^{\circ }}$), our lattice angles (Table S3) deviate around 1 %, which indicate triclinic-like behavior according to the absence of temperature effects. However, our results suggest a triclinic-like $\rightarrow$ orthorhombic-like transition as the number of layers increases (n = $1 \rightarrow 5$), which is expected given the high orthorhombic bulk stability for $T < {163}\hbox { K}$^22^.

**Figure 3:**
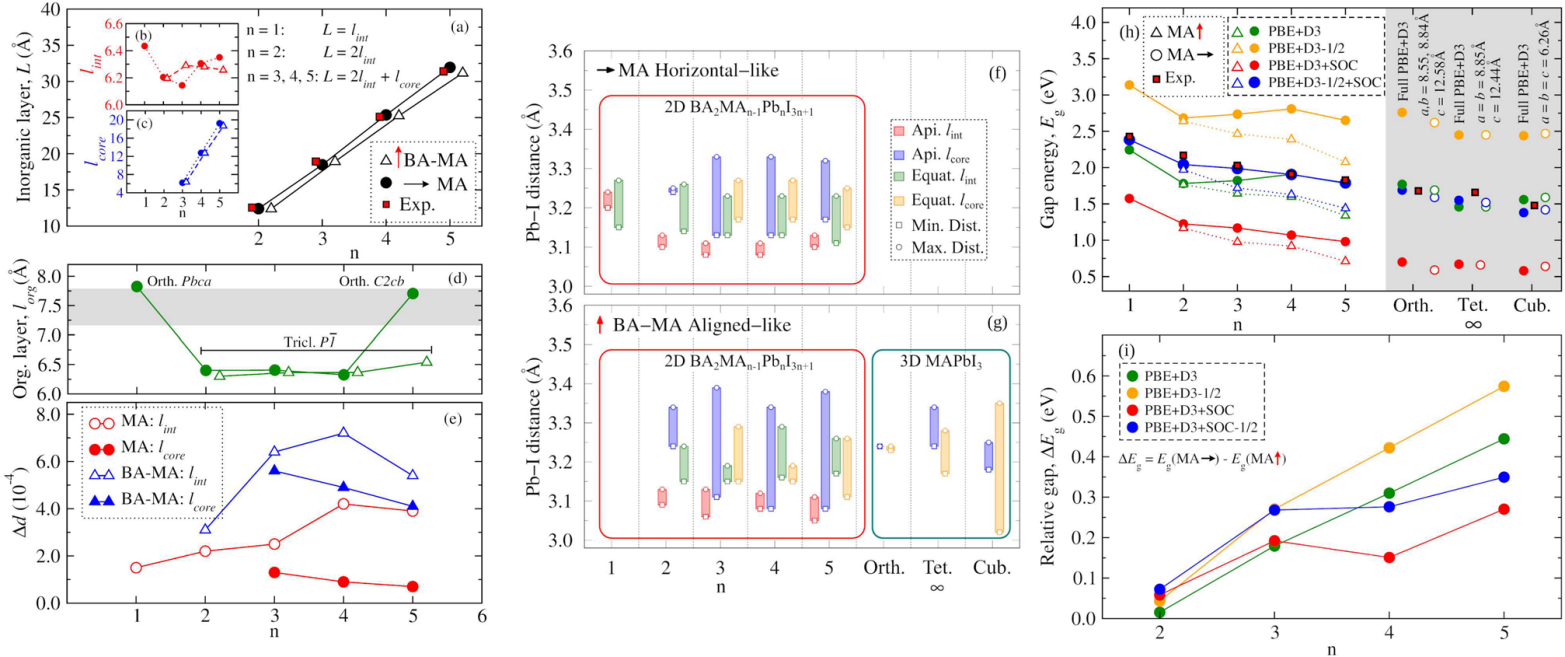
Thickness characterization of inorganic and organic layers through (**a**) *L* (complete ${\hbox {Pb}_{n}\hbox {I}_{3n+1}}$), (**b**) $l_\text {int}$ (PbI$_{6}$ octahedrons at interface region), (**c**) $l_\text {core}$ (PbI$_{6}$ octahedrons at inorganic core region), and (**d**) $l_\text {org}$ (organic BA$_{2}$ width region). The red square symbols and shaded region are—respectively—the *L* and $l_\text {org}$ interval values experimentally reported^12,13^. (**e**) Bond length distortion ($\Delta d$) for the ${\hbox {Pb}_{n}\hbox {I}_{3n+1}}$ layer at the interface and core regions. (**f**) MA horizontal-like and (**g**) BA-MA aligned-like values are depicted separately for the interface ($l_\text {int}$) and core ($l_\text {core}$) regions. (**h**) Band gap energies for MA horizontal-like and BA-MA aligned-like for 2D-RP perovskites calculated through the DFT-1/2 and SOC combinations and compared with the experimental values^12,13,22–25,67,68^. The same protocol set was employed for the orthorhombic, tetragonal, and cubic bulks fully relaxed and for relaxed atomic positions into experimental lattice parameters; see the values indicated. (**i**) Relative gap calculated as $\Delta E_\text {g} = E_\text {g}(\text {MA}\rightarrow ) - E_\text {g}(\text {MA}\uparrow )$ between the MA horizontal-like and BA-MA aligned-like values for n = 2–5.

Figure 3 depicts the *L*, $l_\text {int}$, $l_\text {core}$, and $l_\text {org}$ concerning the thickness of the inorganic layer, interface octahedrons, core octahedrons, and organic layer as shown in Fig. 1. Panel (a) shows that our *L*(n = 1–5) values (linearly correlated with n) for both MA horizontal-like and BA-MA aligned-like configurations are in excellent agreement with 2D-RP perovskites at room temperature^12,13^. The *L*(n) correlation is due to the linear $L = l_\text {int}$ (n = 1), $L = 2l_\text {int}$ (n = 2), and $L = 2l_\text {int} + l_\text {core}$ (n = 3–5)—panels (b) and (c)—behavior. However, between n = 3 and n = 5 the $l_\text {int}$ values reveal the effects of local octahedral distortions as a result of interface stress between inorganic and organic layers. Panel (d) shows that the organic layer width for n = 1 and 5, with MA horizontal-like, lies into the interval (shaded region) reported by experimental works for orthorhombic phases (*Pbca* and *C*2*cb*)^12,13,66^, whereas for n = 2–4 (in both configurations) and n = 5 (only for BA-MA aligned-like) it is in triclinic phase, being $l_\text {org}$ slightly compressed compared to the orthorhombic phase.

The bond length distortions ($\Delta d$)—panel (e)—calculated for MA horizontal-like are in agreement with experimental results for ${\hbox {BA}_{2}\hbox {MA}_{n-1}\hbox {Pb}_{n}\hbox {I}_{3n+1}}$ between n = 2 and n = 4 at 250 K^15^. One observes a tendency towards zero convergence (or increasing with n) for $\Delta d$ to $l_\text {core}$ ($l_\text {int}$), which indicates a centrosymmetric behavior similar to bulk one (especially for tetragonal and orthorhombic, Table S5 in Supporting Information and Ref.^29^) for the core region as the inorganic layer increases with n. Conversely, for BA-MA aligned-like $\Delta d$ is more pronounced, even though $\Delta d(l_\text {int}) > \Delta d(l_\text {core})$ is kept, evidencing that the initial BA-MA alignment yields distortions not only in the organic-inorganic interfaces, but also in the core region. Bond angle variance ($\sigma ^2$)—Table S4—in MA horizontal-like for interface and core follows the same tendency as for $\Delta d$, i.e., $\sigma ^2(l_\text {int}) > \sigma ^2(l_\text {core})$. However, in BA-MA aligned-like is inverse, so that $\sigma ^2(l_\text {int}) < \sigma ^2(l_\text {core})$ indicates a mutual angular distortion by MA at the cuboctahedral site with BA organic spacers.

The regional distortions from $\Delta d$ concerning the non-centrosymmetric octahedral profile were locally detailed through the Pb–I distances, i.e., calculated for the equatorial region and apical direction at the interface and core parts of the inorganic layer. Figure 3 shows the Pb–I distances for the equatorial plane and apical direction for both regions considering MA horizontal-like—panel (f)—and BA-MA aligned-like—panel (g)—configurations, as well as for the bulks: orthorhombic, tetragonal, and cubic. For n = 1, the Pb–I distances indicate that the non-centrosymmetric profile is more pronounced in the equatorial region, whereas for the n = 2–5 interval, the interaction between the octahedrons and the MA cations yields a high metal displacement (as a ferroelectric metal off-centering configuration) in the apical direction of the octahedrons in the core region. However, it is observed that the BA-MA aligned-like configuration is more non-centrosymmetric than the MA horizontal-like, which indicates that the initial BA-MA alignment yields octahedrons in which the metal off-centering profile is more pronounced. In the next sections, we show how this difference for the Pb–I distances between MA horizontal-like and BA-MA aligned-like about metal off-centering profile impacts not only on energy gap values (given the partially canceled dipole moments, $\uparrow {\mu }\text {(MA)}\cdot \downarrow {\mu }\text {(BA)}$), but also other important optoelectronic properties.

### Optoelectronic analysis

#### Energy gap with DFT+D3+SOC-1/2

We calculated the band gap energies ($E_\text {g}$) for ${\hbox {BA}_{2}\hbox {MA}_{n-1}\hbox {Pb}_{n}\hbox {I}_{3n+1}}$ 2D-RP perovskites considering MA horizontal-like and BA-MA aligned-like configurations as well as 3D bulks (e.g., orthorhombic, tetragonal, and cubic) through several calculation protocols, such as: PBE+D3, PBE+D3-1/2, PBE+D3+SOC, and PBE+D3+SOC-1/2. Figure 3h shows the $E_\text {g}$ values for the bulks, and as expected from previous studies for 3D halide perovskites^29,30,48^, the results obtained through PBE+D3+SOC-1/2 reached an excellent agreement with the experimental values. For instance, MAPbI$_{3}$ fully relaxed through PBE+D3 as orthorhombic, cubic, and tetragonal presented band gap energies (and deviation from experiments) of $E_\text {g} = {1.68}$ (0.7 %^23,67^), 1.66 (2.0 %^23,24^), and ${1.50}\hbox { eV}$ (2.4 %^22,25,68^), respectively. Furthermore, to investigate the impact of the optimization protocol involving PBE including vdW correction, we performed the relaxation of the atomic positions keeping the experimental lattice parameters fixed (see the structural parameters in Table S5 of Supporting Information), which yielded $E_\text {g}$ deviations less than 11 % (for cubic). Therefore, since PBE+D3-1/2 overestimates and PBE+D3+SOC underestimates $E_\text {g}$, as well as PBE+D3 lies very close to the experimental results as the well-known fortuitous error compensation^11,69^, our PBE+D3+SOC-1/2 protocol is quite suitable for gauging $E_\text {g}$ of the 2D/3D systems investigated here.

For 2D-RP, the PBE+D3+SOC-1/2 protocol employed in the MA horizontal-like configuration also yielded the best $E_\text {g}$(deviation from experiments) values of 2.39 (–0.83 %), 2.04 (–5.9 %), 1.98 (–2.06 %), 1.90 (–0.24 %), and 1.79 (–2.18 %) for n = 1, 2, 3, 4, and 5, respectively^12,13^. The relative gap energies as $\Delta E_\text {g} = E_\text {g}(\text {MA}\rightarrow ) - E_\text {g}(\text {MA}\uparrow )$, i.e., as a difference between the MA horizontal-like and BA-MA aligned-like gap energy values for n = 2–5, were calculated, as depicted in Fig. 3i. The $\Delta E_\text {g}$ values are minimal to all protocols, however, it increases linearly by n > 2 for PBE+D3 and PBE+D3-1/2 protocols, from which one realizes the role of the SOC correction joint with the new lines of MA, so that, from n = 3, $\Delta E_\text {g}$ seems to be constant. The overestimated (PBE+D3-1/2) and underestimated (PBE+D3+SOC) $E_\text {g}$ behavior for both MA horizontal-like and BA-MA aligned-like configurations are similar with respect to the bulks, so that the PBE+D3 calculations reflect for n = 4 and 5 the $E_\text {g}$ values according to the experimental values by compensating the observed errors for 3D MAPbI$_{3}$, which can be attributed to the larger width of the inorganic layer closer to the bulk-like behavior. However, all protocols suggest that small deviations for the calculated $E_\text {g}$ values are kept only for MA horizontal-like configurations throughout n = 1–5, whereas for BA-MA aligned-like the partially canceled one involving the MA and BA dipole moments yields the band gap energies closing, especially from n = 3.

**Figure 4:**
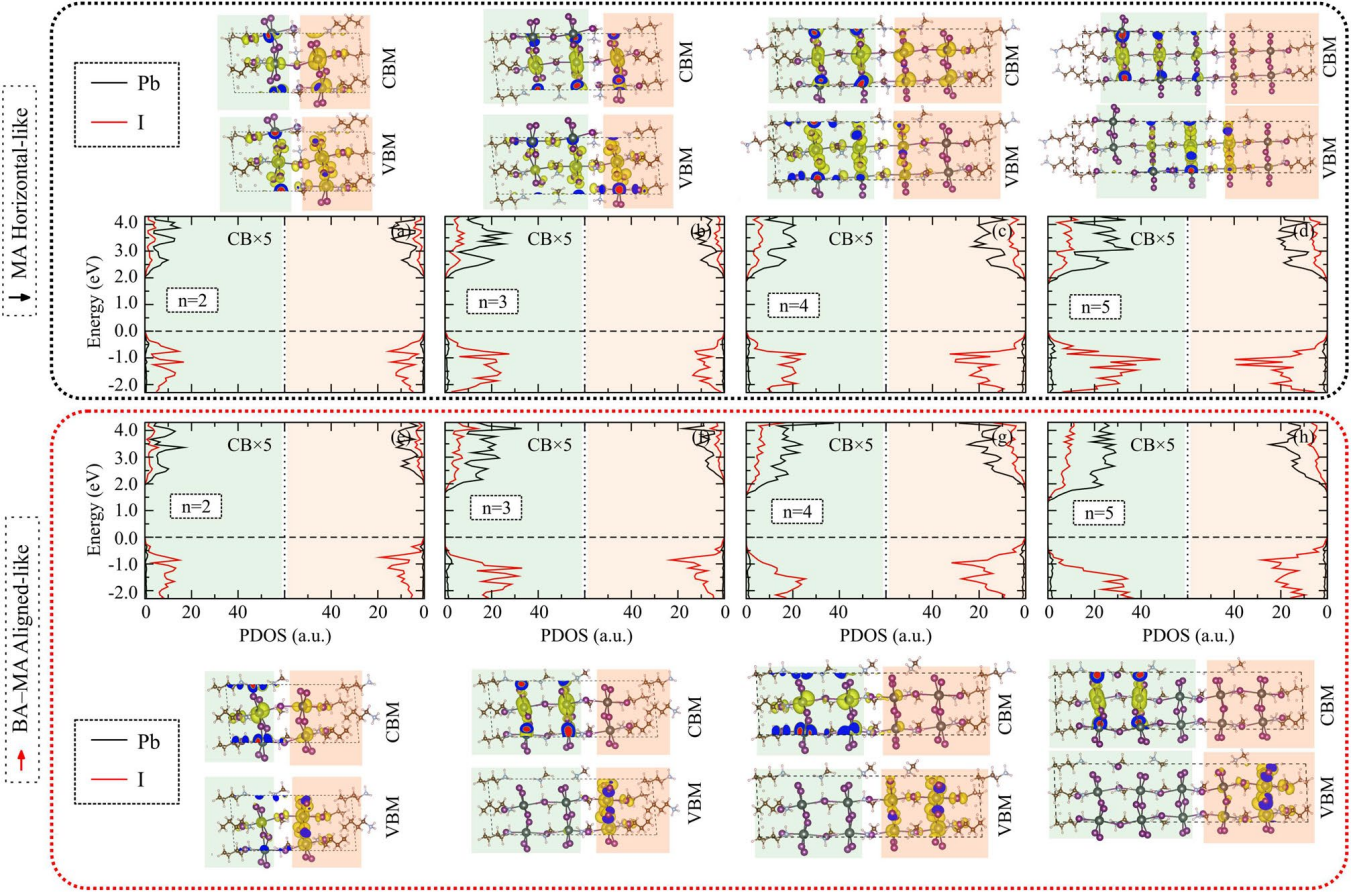
Projected density of states (PDOS) in the respective planes indicated by the corresponding blue and pink colors for the shaded regions in ${\hbox {BA}_{2}\hbox {MA}_{n-1}\hbox {Pb}_{n}\hbox {I}_{3n+1}}$ for MA horizontal-like, from (**a**) to (**d**), and BA-MA aligned-like, from (**e**) to (**h**). Orbital representations in VBM and CBM are above and below the PDOS for MA horizontal-like and BA-MA aligned-like configurations, respectively. All the calculations were performed through PBE+D3+SOC-1/2 protocol.

The $E_\text {g}$ closing magnitude observed through MA horizontal-like $\rightarrow$ BA-MA aligned-like, obtained via PBE+D3+SOC-1/2, is correlated with the number of layer, e.g., $E_\text {g} = {2.04}\hbox { eV} \rightarrow {1.97}\hbox { eV}$ for n = 2, $E_\text {g} = {1.98}\hbox { eV} \rightarrow {1.71}\hbox { eV}$ for n = 3, $E_\text {g} = {1.90}\hbox { eV} \rightarrow {1.63}\hbox { eV}$ for n = 4, and $E_\text {g} = {1.79}\hbox { eV} \rightarrow {1.44}\hbox { eV}$ for n = 5. The same behavior trend is observed for the other protocols. The relative energies comparing the MA horizontal-like and the BA-MA aligned-like configurations, i.e., $E_\text {rel} = E_{\textrm{BA}-\textrm{MA}} - E_{\textrm{MA}}$ calculated through PBE+D3+SOC protocol, point to MA horizontal-like as more stable by 0.79 eV/atom for n = 2, 1.51 eV/atom for n = 3, 2.18 eV/atom for n = 4, and 1.37 eV/atom for n = 5, which suggests that even under finite temperature MA horizontal-like dominates in the 2D-RP material by considering the $E_\text {g}$ values reported by experiments.

#### Projected density of states

Based on previous results, we investigated the suppression effect of the partial cancellation of the MA-BA dipole moments over the PbI inorganic layer as the origin of the $E_\text {g}$ closing. To provide an electronic causality for these observations, Fig. 4 depicts the projected density of states (PDOS) calculated using PBE+D3+SOC-1/2 for each ${\hbox {BA}_{2}\hbox {MA}_{n-1}\hbox {Pb}_{n}\hbox {I}_{3n+1}}$ 2D-RP from n = 2 up to 5 for both MA horizontal-like and BA-MA aligned-like. The PDOS were projected onto the lead (black lines) and iodine (red lines) atoms belonging to the planes perpendicular to the *ab*-plane, which were separated concerning both interface regions between inorganic (${\hbox {MA}_{n-1}\hbox {Pb}_{n}\hbox {I}_{3n+1}}$) and organic (BA$_{2}$) layers. Also for 2D-RP, it is clear the majority (minority) contribution of the I-*p* (Pb-*s*) to valence band maximum (VBM), whereas VBM is dominated by the Pb-*p* orbitals. The VBM and conduction band minimum (CBM) are plotted with their respective orbitals also depicted, on which the atoms in the highlighted blue and pink shaded regions correlate with the plots around the Fermi level (at the VBM).

We found that the partial MA alignment with each other, i.e., $\overrightarrow{\mu }\text {(MA)}\cdot \downarrow {\mu }\text {(BA)}$ in the MA horizontal-like configuration, yields the spreading of their respective charge densities in the ${\hbox {MA}_{n-1}\hbox {Pb}_{n}\hbox {I}_{3n+1}}$ sublattice independent of n, since electrons are equally attracted by MA cations into all cuboctahedral cavities. As a consequence, the I-*p* and Pb-*p* orbital contributions by concerning both interface regions are degenerate, as shown in panels (a) to (d), which explains the agreement between the $E_\text {g}$ experimentally measured and $E_\text {g}$ theoretically estimated values. On the other hand, partially suppressing the dipole moment through $\uparrow {\mu }\text {(MA)}\cdot \downarrow {\mu }\text {(BA)}$ in the BA-MA aligned-like configurations, we found a degeneracy breaking in the PDOS profile between opposing interface planes highlighted by blue and pink colors so that VBM dominates one inorganic-organic interface. In contrast, CBM dominates the opposite ones, as reflected by the orbital representations. For n = 1, the partial dipole moment cancellation involves only the BA cations on the opposite side of the PbI$_{4}$ layer, since the ${{\hbox {NH}_{3}}^{+}}$ terminal is not aligned on the opposite BA cations side (see Fig. S3 in Supporting Information). However, this behavior is slightly suppressed for n = 2, so the alignment is partially as $\uparrow {\mu }\text {(MA)}\cdot \downarrow {\mu }\text {(BA)}$, whereas for the opposite interface the BA cations are set as $\uparrow {\mu }\text {(MA)}\cdot \uparrow {\mu }\text {(BA)}$. Thus, by increasing the inorganic layer width by adding the PbI and MA species, this suppression is highly pronounced for n = 3–5, in which the partial cancellation of the dipole moments is more effective. Therefore, our result set demonstrates that the energy gap closure is due to the intercalated downward shift to Pb-*p* at the CBM from the blue planes (see also Fig. S4), while the VBM is maintained as our Fermi level through the I-*p* orbitals (apical and equatorial sites) at the opposite octahedral interfaces.

#### Bulk Rashba splitting effect

**Figure 5:**
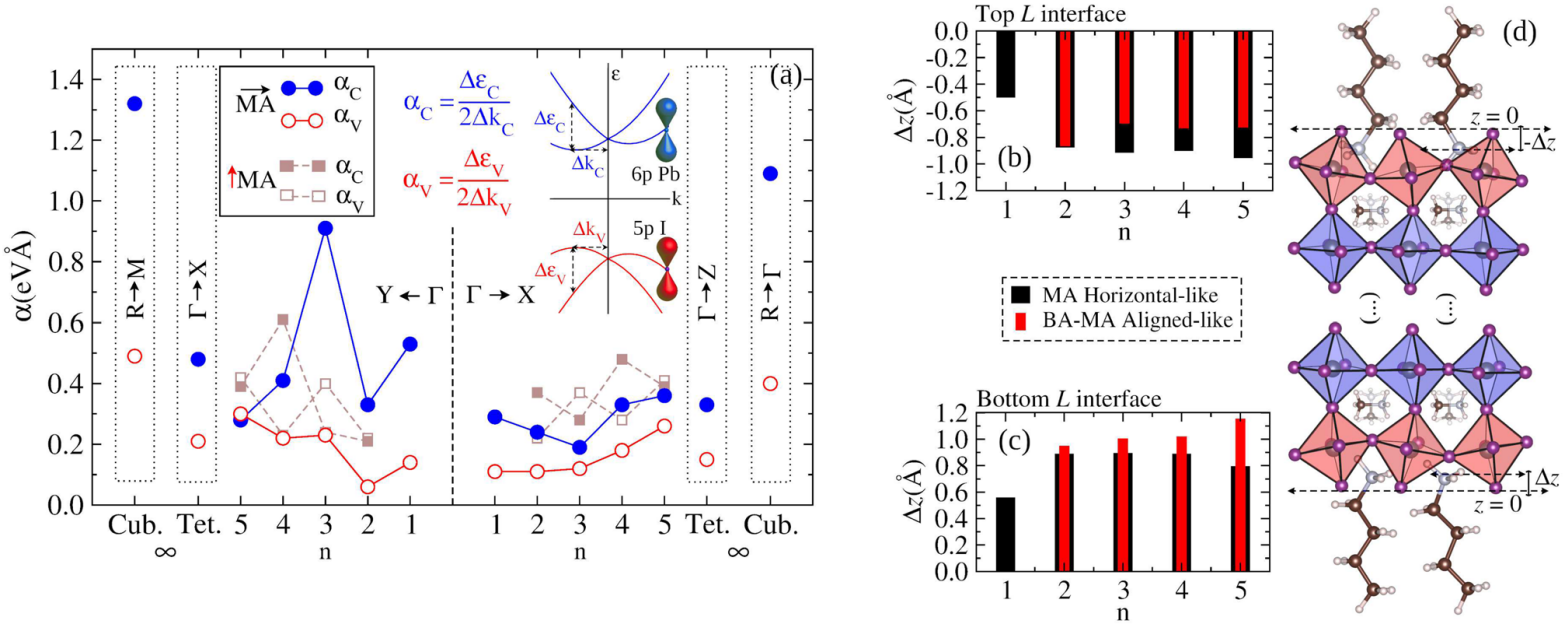
(**a**) Rashba splitting for parabolic bands throughout $\text {Y} \leftarrow \Gamma$ and $\Gamma \rightarrow \text {X}$ branches for VBM ($\alpha _\text {V}$) and CBM ($\alpha _\text {C}$) for ${\hbox {BA}_{2}\hbox {MA}_{n-1}\hbox {Pb}_{n}\hbox {I}_{3n+1}}$ with respect to n layers and for orthorhombic, tetragonal, and cubic bulks (from paths indicated in dotted rectangles). The schematic representation of Rashba splitting and its definition as $\alpha = \Delta \varepsilon /2\Delta k$ used in the calculation. Penetration degree ($\Delta z$) of BA spacers in the ${\hbox {Pb}_{n}\hbox {I}_{3n+1}}$ inorganic layer for the top (**b**) and bottom (**c**) interfaces based on the scheme in (**d**). $\Delta z$ for both MA horizontal-like and BA-MA aligned-like configurations were calculated conventionally by taking $\Delta z < 0$ for top and $\Delta z > 0$ for bottom.

We quantified the bulk Rashba splitting for the valence and conduction bands for the ${\hbox {BA}_{2}\hbox {MA}_{n-1}\hbox {Pb}_{n}\hbox {I}_{3n+1}}$ (with respect to n) and bulk systems through the Rashba coefficient ($\alpha = \Delta \varepsilon /2\Delta k$)^29,34,70^, Fig. 5. $\alpha _\text {C,V}$ are computed by the eigenvalues difference ($\Delta \varepsilon$) relative to the $\varepsilon$ wells vertices at a particular *k*-point ($\Delta k$) from $\Gamma$ point for the 2D-RP (see band structures in Fig. S5 in Supporting Information). In line with previous works for MABI$_{3}$ (B = Si, Ge, Sn, and Pb)^26,29,32,34,70^, the bulk Rashba splitting emerges from a strong SOC energy portion associated with the metal off-centering contributions within the octahedrons. For instance, $\alpha _\text {C,V}$ decreases to the cubic $\rightarrow$ tetragonal transition, moving to $\alpha _\text {C,V} = {0}\hbox { eV }$Å for orthorhombic, which is associated with increased metal on-centering on this sequence of structures. The same behavior is observed for 2D-RP concerning the correlation between $\alpha _\text {C,V}$ and metal off-centering magnitude, given by $\alpha _\text {V}$ and $\alpha _\text {C}$ and calculated for VBM and CBM along the Brillouin zone paths, as indicated, e.g., $\text {Y} \leftarrow \Gamma$ and $\Gamma \rightarrow \text {X}$ branches for n = 2–5 for MA horizontal-like and BA-MA aligned-like configurations. However, comparatively taking both MA horizontal-like and BA-MA aligned-like configurations and even the presence of interfaces, the Rashba parameters for 2D-RP are more complex, mainly due to the role of organic spacers on apical iodines in the interface region.

MA horizontal-like and BA-MA aligned-like configurations differ by concerning the relative $\alpha _\text {V}$ and $\alpha _\text {C}$ values, from which $\alpha _\text {C} > \alpha _\text {V}$ for all number of layers of the MA horizontal-like (including n = 1). For BA-MA aligned-like configurations, the same is observed only for n = 2 and 4, while for n = 3 and 5 one observes $\alpha _\text {C} < \alpha _\text {V}$. As observed, the Pb–I distance results in Fig. 3 indicate for n = 3 and 5 in BA-MA aligned-like configurations a higher metal off-centering (for $l_\text {int}$) than in MA horizontal-like configurations. This result correlates with the inversion through $\alpha _\text {C} < \alpha _\text {V}$ with respect to the MA horizontal-like findings, given that for BA-MA aligned-like configurations the Pb–I distances suggest that apical iodines at the organic-inorganic interface suffer more stress compared to other ones within the ${\hbox {Pb}_{n}\hbox {I}_{3n+1}}$ inorganic layer.

We advanced in the interface characterization by describing both organic-inorganic interfaces, which can be detailed through the penetration degree ($\Delta z$) of the BA spacers in the ${\hbox {Pb}_{n}\hbox {I}_{3n+1}}$ inorganic part. $\Delta z$ was similarly calculated through equation of $l_\text {org}$ depicted in Fig. 2, however, by taking the distance between the plane formed by apical low-coordinated iodines at the interface and the ${{\hbox {NH}_{3}}^{+}}$ group at the BA tail (see the details in Supporting Information). $\Delta z$ allows correlating the proximity between ${{\hbox {BA-NH}_{3}}^{+}}$ tail and the apical iodine at the interface, consequently suggesting its impact on the PDOS and Rashba splitting. Figure 5 depicts the calculated $\Delta z$ values for the MA horizontal-like and BA-MA aligned-like configurations by taking top-panel (a)—and bottom-panel (b)—interfaces, which were considered respectively as $z = 0 \rightarrow -\Delta z$ and $z = 0 \rightarrow \Delta z$. It is observed that from $\text {n} = 1 \rightarrow 2, 3, 4$, and 5 the penetration degree increases, but while remaining similar to the top and bottom interfaces for MA horizontal-like, given the dipole moments as $\overrightarrow{\mu }\text {(MA)}\cdot \downarrow {\mu }\text {(BA)}$, for BA-MA aligned-like $\Delta z$ at the top interface it is smaller than at the bottom. This is explained by the repulsion involving the ${{\hbox {NH}_{3}}^{+}}$ groups from MA and BA partially opposite at the top, so that the dipole moment $\uparrow {\mu }\text {(MA)}\cdot \downarrow {\mu }\text {(BA)}$, whereas on the bottom interface is $\uparrow {\mu }\text {(MA)}\cdot \uparrow {\mu }\text {(BA)}$, which in turn results in $\Delta z$ large. This behavior correlates with the electron depletion observed through PDOS at one of the interfaces from which the opposite interface forms the VBM in BA-MA aligned-like configurations. Additionally, the shortest $\Delta z$ value for $\uparrow {\mu }\text {(MA)}\cdot \downarrow {\mu }\text {(BA)}$ interface means that BA spacers are closer to the apical iodine (which is the majority for VBM), yielding additional stress on the interface octahedrons, resulting in the behavior observed as $\alpha _\text {C} < \alpha _\text {V}$ for Rashba splitting at CBM and VBM.

#### Optical absorption coefficient

**Figure 6:**
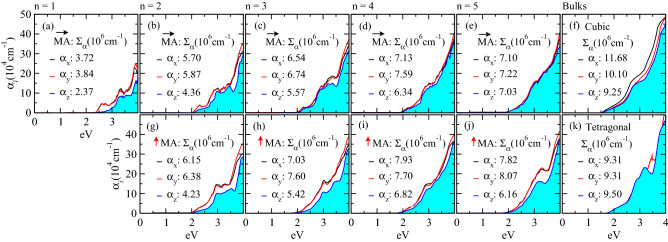
Absorption coefficients, $\alpha (\omega )$, calculated via DFT+D3+SOC-1/2 for all ${\hbox {BA}_{2}\hbox {MA}_{n-1}\hbox {Pb}_{n}\hbox {I}_{3n+1}}$ 2D-RP through the MA horizontal-like (**a**)–(**e**) and BA-MA aligned-like (**g**)–(**j**) configurations, given that *x* ($\alpha _x$), *y* ($\alpha _y$), and *z* ($\alpha _z$) directions were considered separately. $\alpha _z$ is filled for all 2D-RP systems, as well as for the cubic (**f**) and tetragonal (**k**) bulks. Total absorbance ($\sum _{\alpha }$) values are depicted for the 0.0–4.2 eV interval.

Advancing in a deeper description of the ${\hbox {BA}_{2}\hbox {MA}_{n-1}\hbox {Pb}_{n}\hbox {I}_{3n+1}}$ 2D-RP and bulks like cubic and tetragonal (see for orthorhombic in Fig. S6 in Supporting Information), we compared the absorption coefficients with respect to the *x* ($\alpha _x$), *y* ($\alpha _y$), and *z* ($\alpha _z$) directions among MA horizontal-like and BA-MA aligned-like configurations, as shown in Fig. 6. The UV-Vis spectra were calculated by computing the imaginary and real parts of the dielectric function of the systems. The former was obtained within the random phase approximation (RPA)^71^ and the latter through a Kramers-Kronig transformation^72,73^. For bulks, the absorption coefficients isotropy can be seen through the highest/lowest quotient, which are $\alpha _x/\alpha _y = 1.27$, $\alpha _x/\alpha _z = 1.02$, and $\alpha _y/\alpha _x = 1.09$ for cubic, tetragonal, and orthorhombic, respectively. For 2D-RP configurations one observes that $\alpha _x \approx \alpha _y$ over the entire energy range, while $\alpha _x (\alpha _y) > \alpha _z$ for which the confinement is pronounced, especially for n = 1 and 2. On the other hand, the anisotropic behavior from the confinement throughout the *z* direction is suppressed in MA horizontal-like as the number of layers increases, as well as highlighted by the total absorbance ($\sum _{\alpha }$), so that $\alpha _x \sim \alpha _y \sim \alpha _z$ for n = 5. For instance, while the $\alpha _y/\alpha _z$ quotient for n = 1 is 1.62, it keeps decreasing as $1.34 \rightarrow 1.21 \rightarrow 1.19 \rightarrow 1.04$ for n = $2 \rightarrow 3 \rightarrow 4 \rightarrow 5$ in MA horizontal-like configurations, which suggest a convergence for a bulk-like behavior as observed on tetragonal and orthorhombic structures. Conversely, for BA-MA aligned-like configurations the anisotropy remains as the number of layers increases, so that $\alpha _y/\alpha _z = 1.51 \rightarrow 1.40 \rightarrow 1.16 \rightarrow 1.31$ for n = $2 \rightarrow 3 \rightarrow 4 \rightarrow 5$. Thus, we found that the relative orientation involving the organic spacers and cations that hardly can be controlled in the experiment plays a moderate role in the 2D-RP $\rightarrow$ bulk convergence for optical properties. However, a favorable MA horizontal-like configuration converges to the bulk absorption coefficient and can keep the performance of 2D-RP comparable to 3D perovskite.

## Summary

We carried out a systematic and rigorous investigation from first-principles calculations of the ${\hbox {BA}_{2}\hbox {MA}_{n-1}\hbox {Pb}_{n}\hbox {I}_{3n+1}}$ 2D Ruddlesden-Popper perovskites properties. We considered the MA horizontal-like and BA-MA aligned-like configurations (with relative dipole moment orientations as $\overrightarrow{\mu }\text {(MA)}\cdot \downarrow {\mu }\text {(BA)}$ and $\uparrow {\mu }\text {(MA)}\cdot \downarrow {\mu }\text {(BA)}$, respectively) throughout n = 1, 2, 3, 4, and 5 number of inorganic layers, following a detailed structural description within the DFT+vdW+SOC protocol. The set of results based on organic cation and spacer configurations is able to indicate the correct relative orientation according to the relative dipole moments, reaching the structural and optoelectronic parameters in close correlation with experiments. For instance, we employed the DFT-1/2 quasiparticle correction (as a low-cost method) used for the 2D-RP perovskites to predict the optoelectronic properties, yielding new insights given the agreement with the experimental results. Our results indicate that relative organic spacers and cation orientations promote linear growth of the ${\hbox {Pb}_{n}\hbox {I}_{3n+1}}$ inorganic layer width as n increases, which is determined uniquely by the core region dimensions. However, the organic spacers and cation configurations yield not only different local distortions in the core and interface octahedrons (in ${\hbox {Pb}_{n}\hbox {I}_{3n+1}}$ sublattice) but also different compression patterns of the organic spacers layer—also their penetration magnitude at the inorganic layer interface—according to the synthesis of orthorhombic (*Cc*2*m* and *Cmca*) and triclinic ($P{\bar{1}}$) phases at low temperatures. DFT+D3+SOC-1/2 shed light on MA horizontal-like configuration as the best relationship between calculated and experimental band gap energy for all 2D-RP perovskites. This result suggests that, on average, as the most stable configuration, it is the majority in the ${\hbox {BA}_{2}\hbox {MA}_{n-1}\hbox {Pb}_{n}\hbox {I}_{3n+1}}$ optoelectronic properties. Furthermore, we highlighted the role of partially canceled dipole moments involving ${{\hbox {NH}_{3}}^{+}}$ terminal groups in BA-MA aligned-like configurations, energy gap closing through an electron depletion mechanism as a consequence of the intercalation downward shift for the Pb-*p* (CBM) relative to the I-*p* (VBM) orbitals at the band edges. Furthermore, the anisotropy $\rightarrow$ isotropy optical absorption conversion as the layer number increases (as a bulk convergence) appears only for the MA horizontal-like configuration, which correlates with the band gap energies in agreement with the experimental results. Finally, we observed that the high distortion degree in the interface octahedrons for the BA-MA aligned-like configuration promotes additional stress of the BA spacers on the apical iodine at the interface, resulting in the $\alpha _\text {C} < \alpha _\text {V}$ inverse behavior (at CBM and VBM) for Rashba splitting with respect to the MA horizontal-like configuration ($\alpha _\text {C} > \alpha _\text {V}$).

## Supplementary Information

Supplementary Information.

## Data Availability

The datasets used and/or analysed during the current study available from the corresponding author on reasonable request.
