# Supplementary material for: Revealing the impact of organic spacers and cavity cations on quasi-2D perovskites via computational simulations
Source: Sci Rep. 2023 Mar 17;13:4446. doi: 10.1038/s41598-023-31220-8 (PMC10023785; doi:10.1038/s41598-023-31220-8)
Supplement: Supplementary file 1 — Supplementary Information. [file 41598_2023_31220_MOESM1_ESM.pdf]

Quasi-2D $BA_2MA_{n-1}Pb_nI_{3n+1}$ Perovskites: The Importance of the Organic Spacers and Cavity Cations Configuration

Diego Guedes-Sobrinho,[*,†] Danilo Neves Silveira,[*,†] Luis O. de Araujo,[*,†] Jônatas Favotto Dalmedico,[*,‡] W. Wenzel,[*,¶] Y. Pramudya,[*,¶] Maurício J. Piotrowski,[*,‡] and Celso R. C. Rêgo[*,¶]

†*Chemistry Department, Federal University of Paraná, 81531-980, Curitiba, Brazil*

‡*Department of Physics, Federal University of Pelotas, PO Box 354, 96010-900, Pelotas, RS, Brazil*

¶*Karlsruhe Institute of Technology (KIT), Institute of Nanotechnology*

Hermann-von-Helmholtz-Platz, 76344, Eggenstein-Leopoldsha fen, Germany

E-mail: guedessobrinho@ufpr.br; daniloneves@ufpr.br; luisaraujo@ufpr.br; ; wolfgang.wenzel@kit.edu; yohanes.pramudya@kit.edu; mauriciomjp@gmail.com; celso.rego@kit.edu

I Gap energy into DFT-1/2 and spin-orbit coupling

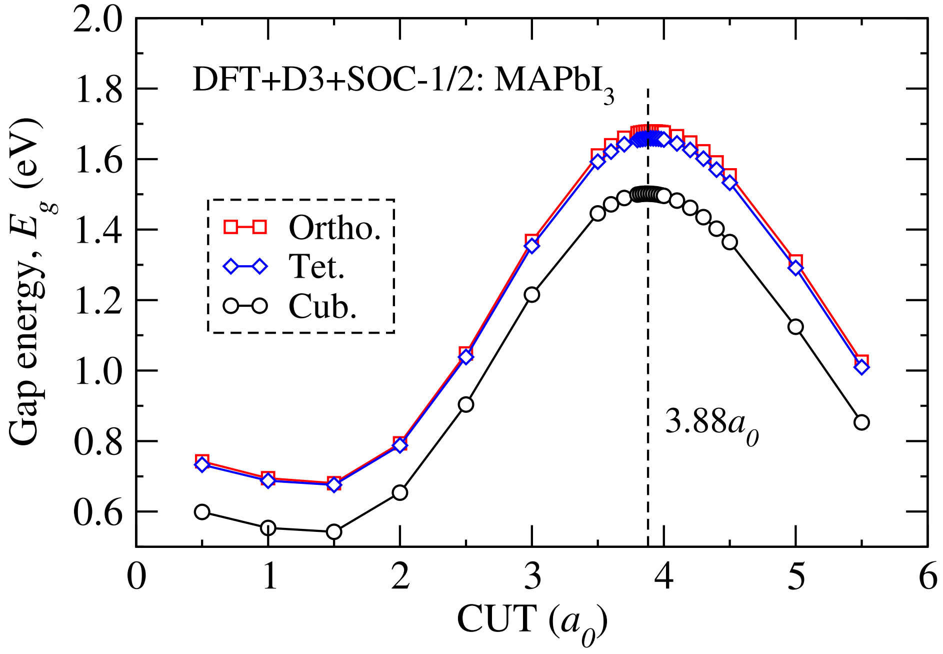

Figure S1 – Optimization of the CUT parameter for $MAPbI_3$ as cubic, tetragonal, and orthorhombic structures, in Bohr radius a_0, within quasiparticle correction by using spin-orbit coupling, i.e., DFT+D3+SOC-1/2, through the half-ionization around the valence band for $5p$ state from the I. The vertical dashed line depicts the good transferability of the method among the different structures, so that the CUT = $3.88a_0$ was used in gap energies calculations for 2D-RP and 3D perovskites.

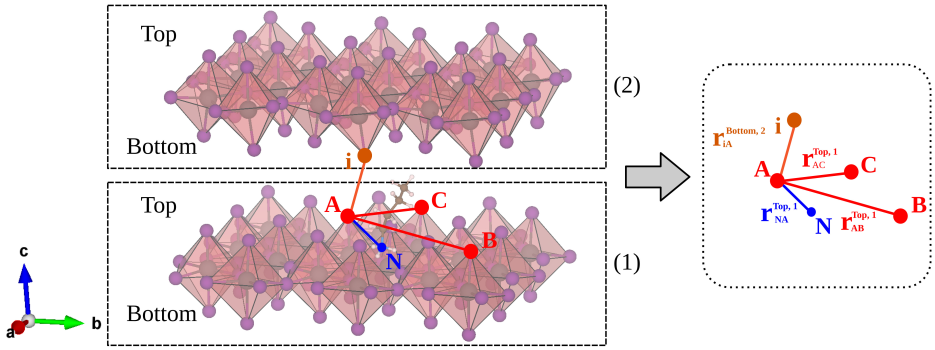

Figure S2 – Variable involved in the plane definition formed by apical low-coordinated iodines for the calculation of the l_{org} and penetration degree (Δz), i.e.,

$$l_{\text{org}} = \frac{|\mathbf{n}\cdot\mathbf{r}_{i,A}^{\text{bottom,2}}|}{\sqrt{n_x^2+n_y^2+n_z^2}}\,, \qquad \Delta z = \frac{|\mathbf{n}\cdot\mathbf{r}_{NA}^{\text{top or botton}}|}{\sqrt{n_x^2+n_y^2+n_z^2}},$$

where n $= (n_x, n_y, n_z)$ **is the normal vector with respect to the plane formed by the apical iodine atoms in the interface region on the top of the inorganic layer, i.e.,** $\mathbf{n} = \mathbf{r}_{AB}^{\text{top,1}} \times \mathbf{r}_{AC}^{\text{top,1}}$**. For** Δz**,** $\mathbf{n} = \mathbf{r}_{AB}^{\text{top,1}} \times \mathbf{r}_{AC}^{\text{top,1}}$ **and** $\mathbf{r}_{NA}^{\text{top or botton}}$ **is the vector position of the N from BA (i.e., from the** NH_3^+ **terminal group) penetrating the inorganic layer.**

II Structural information

Table S1 – Lattice parameters calculated for $(BA)_2(MA)_{n\text{-}1}Pb_nI_{3n+1}$ in MA horizontal-like and BA-MA aligned-like and compared with experiments from literature, e.g., references 1, 2 and 3 which involve Triclinic ($P\bar{1}$) 250 K→ Orthorhombic ($Cc2m$, $Cmca$), 300 K transitions. "?" symbols mean there is no experimental parameter registred, or for n = 1 in which there is no BA-MA aligned-like configuration.

$(BA)_2(MA)_{n\text{-}1}Pb_nI_{3n+1}$: Lattice parameters (Å)									
	Here calculated						Experiments reported from literature		
	MA Horizontal-like			BA-MA Aligned-like			Triclinic ($P\bar{1}$[1]) 250 K→ Orthorhombic ($Cc2m$[2,3], $Cmca$[1]), 300 K		
n	*a*	*b*	*c*	*a*	*b*	*c*	*a*	*b*	*c*
1	8.37	8.92	15.45	?	?	?	?	?	?
2	8.62	9.06	19.39	8.59	9.13	19.35	8.74 → 8.85; 8.85	9.04 → 8.93; 8.94	19.66 → 19.64; 19.67
3	8.72	8.96	25.16	8.65	9.01	25.79	8.78 → 8.86; 8.88	9.02 → 8.93; 8.93	25.43 → 25.98; 25.98
4	8.67	8.90	32.14	8.70	8.96	32.02	8.80 → 8.88; 8.88	8.99 → 8.93; 8.93	31.91 → 32.15; 32.19
5	8.61	8.61	39.93	8.71	9.02	38.04	? → 8.90	? → 8.93	? → 38.52

Table S2 – Volumes calculated for $(BA)_2(MA)_{n\text{-}1}Pb_nI_{3n+1}$ in MA horizontal-like and BA-MA aligned-like and compared with experimental works from literature, e.g., references [1], [2] and [3] which involve Triclinic ($P\bar{1}$) 250 K→ Orthorhombic ($Cc2m$, $Cmca$), 300 K transitions. "?" symbols mean there is no experimental parameter registred, or for n = 1 in which there is no BA-MA aligned-like configuration.

$(BA)_2(MA)_{n\text{-}1}Pb_nI_{3n+1}$: Volume ($Å^3$)			
	Here calculated		Experiments reported from literature
n	MA Horizontal-like	BA-MA Aligned-like	Triclinic,($P\bar{1}$[1]) 250 K → Orthorhombic($Cc2m$[2,3], $Cmca$[1]), 300 K
1	1069.83	?	?
2	1468.97	1471.21	1508.6 → 1552.95
3	1957.29	1976.49	2004.8 → 2057.90
4	2455.03	2473.52	2499.9 → 2551.45
5	3029.72	2963.62	? → 3062.55

Table S3 – Lattice angles calculated for $(BA)_2(MA)_{n\text{-}1}Pb_nI_{3n+1}$ in MA horizontal-like and BA-MA aligned-like and compared with experimental works from literature, e.g., references [1], [2] and [3] which involve Triclinic ($P\bar{1}$) 250 K→ Orthorhombic ($Cc2m$, $Cmca$), 300 K transitions. "?" symbols mean there is no experimental parameter registred, or for n = 1 in which there is no BA-MA aligned-like configuration.

$(BA)_2(MA)_{n\text{-}1}Pb_nI_{3n+1}$: Lattice angles (°)									
	Here calculated						Experiments reported from literature		
	MA Horizontal-like			BA-MA Aligned-like			Triclinic,($P\bar{1}$[1]) 250 K→ Orthorhombic($Cc2m$[2,3], $Cmca$[1]), 300 K		
n	α	β	γ	α	β	γ	α	β	γ
1	111.46	94.10	90.76	?	?	?	?	?	?
2	103.04	95.14	92.37	103.41	94.62	91.56	102.90 → 90; 90	95.09 → 90; 90	91.32 → 90; 90
3	90.51	96.20	92.07	90.96	94.49	91.15	90.03 → 90; 90	95.68 → 90; 90	91.31 → 90; 90
4	98.24	93.49	90.26	97.82	93.13	90.65	97.91 → 90; 90	93.21 → 90; 90	91.22 → 90; 90
5	95.44	91.81	90.03	97.60	91.96	91.13	? → 90	? → 90	? → 90

Table S4 – Largest and shortest tilting angles (°) in equatorial plane and apical directions (Pb–I–Pb) and bond angle variance (σ^2) for I–Pb–I, from which nonzero values indicate distortion from a perfect octahedron characterized by six equal 90° bond angles, so that

$$\sigma^2 = \sum_{i=1}^{12} \frac{(\theta_i - 90)^2}{11},$$

where θ_i are the twelve angles for I–Pb–I on the twelve octahedral quadrants. The "?" correspond to regions without values, e.g., there is no l_{core} for n = 2.

	MA Horizontal-like			BA-MA Aligned-like		
n	Equatorial	Apical	$\sigma^2(l_{int} - l_{core})$	Equatorial	Apical	$\sigma^2(l_{int} - l_{core})$
1	139.35-149.60	?	18.80-?	?	?	?
2	143.23-177.68	146.76-162.68	15.25-?	146.94-175.81	152.04-160.57	15.10-?
3	145.67-176.98	149.27-162.64	17.12-16.99	143.96-174.25	152.64-166.05	22.17-45.25
4	145.89-169.50	165.35-178.11	26.15-13.11	144.48-171.98	159.81-165.00	26.48-43.29
5	142.66-165.15	169.37-177.71	23.48-11.77	145.04-175.95	148.80-162.32	15.23-33.94

Table S5 – Lattice parameters, Pb−I distance (in equatorial and apical directions), and volumes for the $MAPbI_3$ bulks as cubic (Cub.) tetragonal (Tet.), and orthorhombic (Ortho.) fully relaxed through PBE+D3 protocol. Additionally, keeping lattice parameters reported by experimental works – 4, 5, and 6 – we relaxed the atom positions to investigate the impact of the vdW protocol.

Method	Struct.	Lattice parameters			Pb−I(Å)		Volume
		a	*b*	*c*	Equatorial	Apical	$Å^3$
Full PBE+D3	Cub.	6.28	6.23	6.33	3.07-3.23	3.17-3.19	247.89
	Tet.	8.67	8.68	12.85	3.13-3.22	3.19-3.24	967.34
	Orho.	8.34	12.76	8.97	3.18-3.19	3.20-3.20	942.20
Exp.[4–6] a,b,c in PBE+D3	Cub.	6.27	6.27	6.27	3.06-3.23	3.13-3.17	246.49
	Tet.	8.85	8.85	12.44	3.13-3.30	3.10-3.13	974.33
	Orho.	8.55	12.58	8.84	3.17-3.17	3.18-3.19	950.82

III Projected density of states

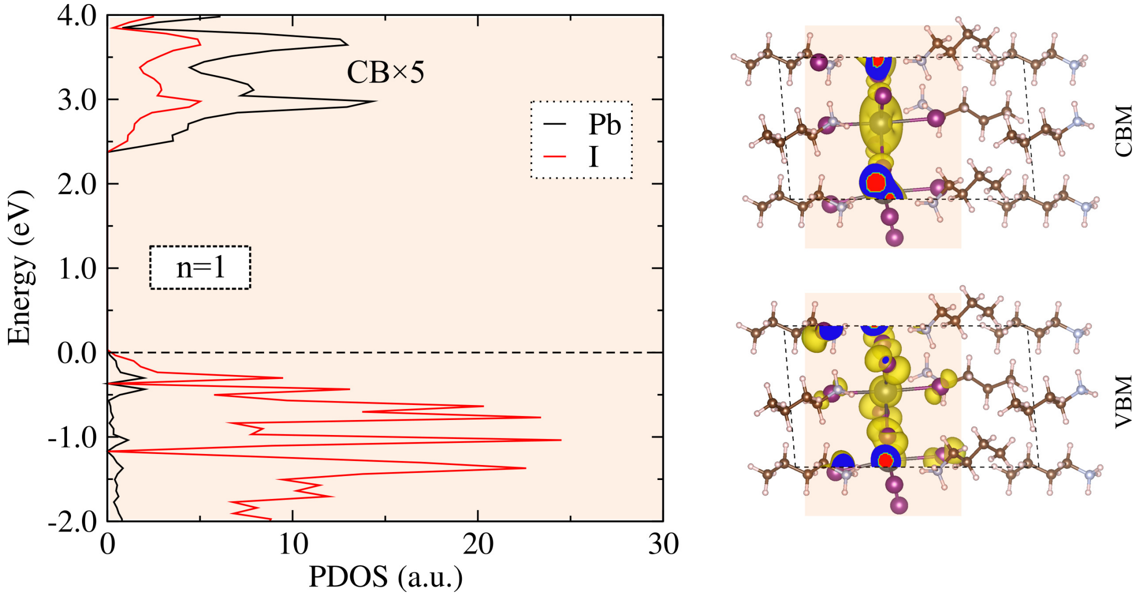

Figure S3 – Projected density of states (PDOS) of Pb and I atoms in inorganic layer (pink shaded regions) in BA_2PbI_4 (n = 1) with the orbital representations at VBM and CBM.

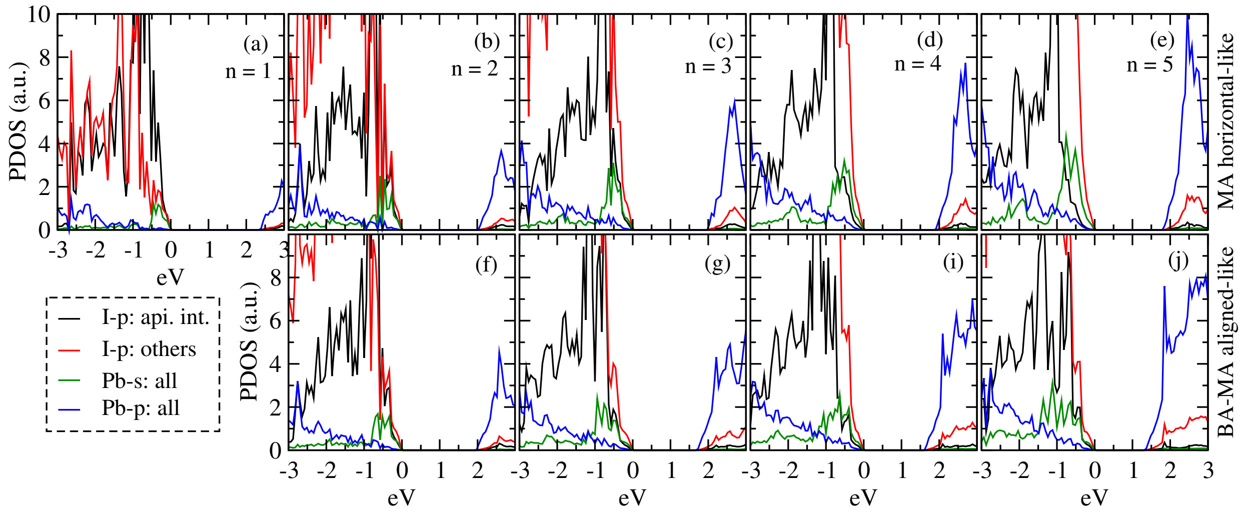

Figure S4 – Projected density of states (PDOS) for $BA_2MA_{n-1}Pb_nI_{3n+1}$ through n = 1, 2, 3, 4, and 5 for MA horizontal-like, from (a) to (e), and BA-MA aligned-like, from (f) to (j). The states were projected by considering p orbitals of I at apical interface sites (I-p: api. int.) and all the others (equatorial and apical in core region, as I-p: others) devided, as well as all Pb-s and Pb-p.

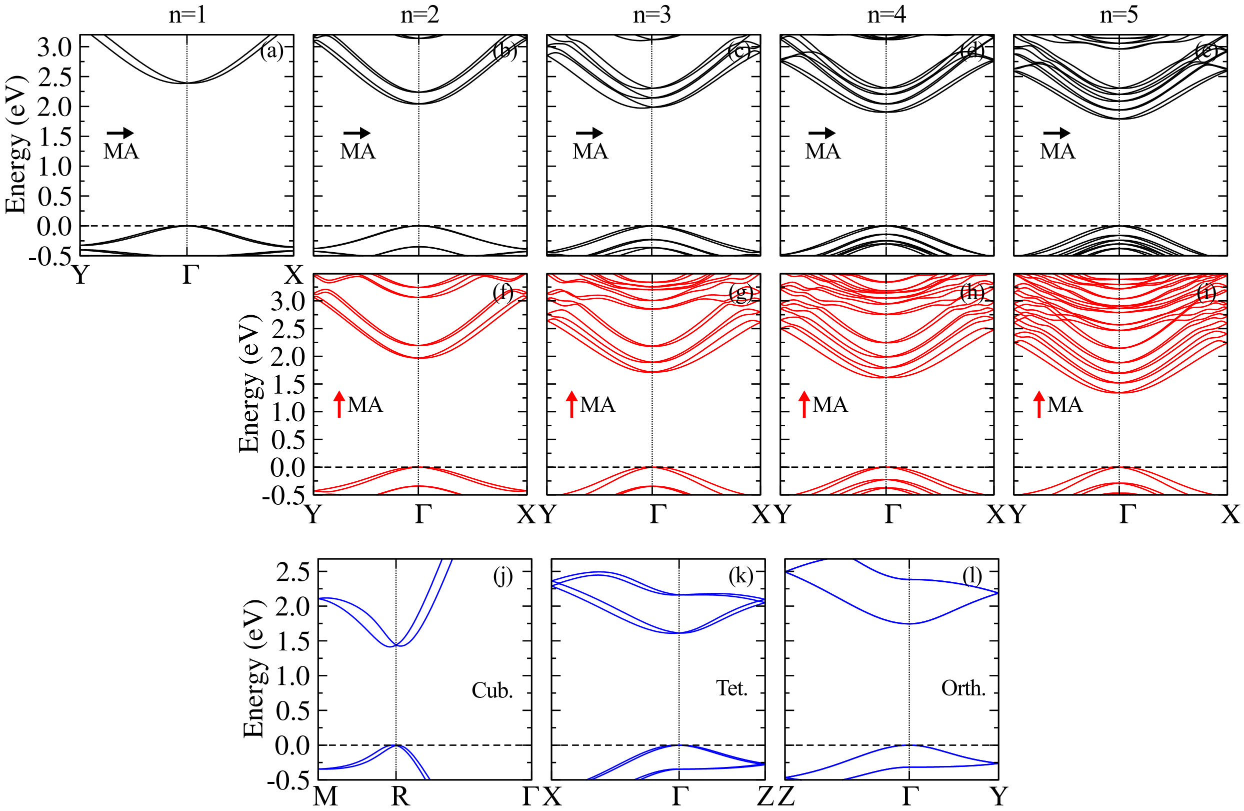

Figure S5 – Band structure for $BA_2MA_{n-1}Pb_nI_{3n+1}$ through n = 1, 2, 3, 4, and 5 for MA horizontal-like, from (a) to (e), and BA-MA aligned-like, from (f) to (i), for Y→ Γ →X. For bulks as cubic (j), tetragonal (k), and orthorhombic (l) M→ R → Γ, X→ Γ →Z, and Z→ Γ →Y, respectively, were considered.

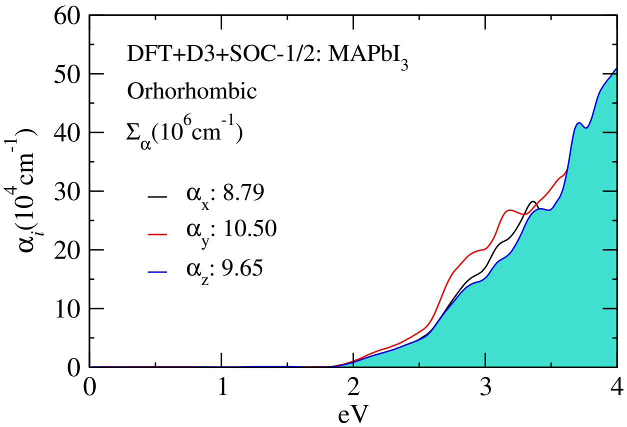

Figure S6 – Absorption coefficients, $\alpha(\omega)$, calculated via PBE+D3-1/2+SOC for bulk as orthorhombic given that x (α_x), y (α_y), and z (α_z) directions were considered separately. α_z is filled and the total absorbance ($\sum_\alpha$) values are depicted for the 0.0-4.2 eV interval.

References

(1) Paritmongkol, W.; Dahod, N. S.; Stollmann, A.; Mao, N.; Settens, C.; Zheng, S.-L.; Tisdale, W. A. Synthetic Variation and Structural Trends in Layered Two-Dimensional Alkylammonium Lead Halide Perovskites. *Chem. Mater.* **2019**, *31*, 5592–5607.

(2) Stoumpos, C. C.; Soe, C. M. M.; Tsai, H.; Nie, W.; Blancon, J.-C.; Cao, D. H.; Liu, F.; Traoré, B.; Katan, C.; Even, J.; Mohite, A. D.; Kanatzidis, M. G. High Members of the 2D Ruddlesden-Popper Halide Perovskites: Synthesis, Optical Properties, and Solar Cells of $(CH_3(CH_2)_3NH_3)_2(CH_3NH_3)_4Pb_5I_{16}$. *Chem* **2017**, *2*, 427–440.

(3) Stoumpos, C. C.; Cao, D. H.; Clark, D. J.; Young, J.; Rondinelli, J. M.; Jang, J. I.; Hupp, J. T.; Kanatzidis, M. G. Ruddlesden–Popper Hybrid Lead Iodide Perovskite 2D Homologous Semiconductors. *Chem. Mater.* **2016**, *28*, 2852–2867.

(4) Baikie, T.; Fang, Y.; Kadro, J. M.; Schreyer, M.; Wei, F.; Mhaisalkar, S. G.; Graetzel, M.; White, T. J. Synthesis and Crystal Chemistry of the Hybrid Perovskite $(CH_3NH_3)PbI_3$ for Solid-State Sensitised Solar Cell Applications. *J. Mater. Chem. A* **2013**, *1*, 5628–5641.

(5) Stoumpos, C. C.; Malliakas, C. D.; Kanatzidis, M. G. Semiconducting Tin and Lead Iodide Perovskites with Organic Cations: Phase Transitions, High Mobilities, and Near-Infrared Photoluminescent Properties. *Inorg. Chem.* **2013**, *52*, 9019–9038.

(6) Hao, F.; Stoumpos, C. C.; Chang, R. P. H.; Kanatzidis, M. G. Anomalous Band Gap Behavior in Mixed Sn and Pb Perovskites Enables Broadening of Absorption Spectrum in Solar Cells. *J. Am. Chem. Soc.* **2014**, *136*, 8094–8099.
